# Anthropometric measurements of the foot cannot predict the screw diameter for fifth metatarsal fractures intramedullary fixation

**DOI:** 10.1007/s00276-023-03267-9

**Published:** 2023-12-06

**Authors:** Panagiotis D. Symeonidis, Trifon Totlis, Iasonas Dermitzakis, Athanasia Papachristodoulou, Ioannis Giatas, Alexandros Beris

**Affiliations:** 1 St Luke’s Hospital, Panorama, Thessaloniki, Greece; 2 Department of Anatomy and Surgical Anatomy, School of Medicine, Faculty of Health Sciences, Aristotle University of Thessaloniki, Thessaloniki, Greece; 3 Thessaloniki Minimally Invasive Surgery (TheMIS) Orthopaedic Center, St Luke’s Hospital, Thessaloniki, Greece; 4 School of Medicine, Faculty of Health Sciences, Aristotle University of Thessaloniki, Thessaloniki, Greece; 5 Affidea Group, Budapest, Hungary; 6 Department of Orthopaedic Surgery, University of Ioannina, 45110 Ioannina, Greece; 7 European Interbalkan Medical Center, Thessaloniki, Greece

**Keywords:** Bone anatomy, Foot anatomy, Anthropometric, Osteometry, Fifth metatarsal fracture, Intramedullary fixation

## Abstract

**Purpose:**

The present study aimed to evaluate the accuracy of anthropometric foot measurements in predicting the diameter of the intramedullary screw for fifth metatarsal fracture fixation. Secondary aim was to identify whether the fifth metatarsal intramedullary canal diameter is correlated to the fifth metatarsal length and the foot dimensions.

**Methods:**

In 29 cadaveric feet, the maximum length of the plantar surface of the foot (PL) and the perimeter of the foot at the level of the fifth metatarsal base (PBFM) were measured using a measuring tape. Subsequently, the fifth metatarsal was excised. Using Computed Tomography scan, the metatarsal length (FML), and the horizontal (HDI) and vertical diameter (VDI) at the isthmus level were measured. The HDI values were grouped in 5 mm increments to correspond to the recommended screw diameter (RSD) for intramedullary fixation. A univariate linear regression analysis considered RSD and HDI as the dependent variables and FML, PL, PBFM as the independent variables. A multivariate regression analysis was performed to examine the predictive value of the two anthropometric measurements. A *p*-value < 0.05 was considered statistically significant.

**Results:**

All six univariate analyses revealed that the dependent variable was significantly correlated with the independent variable. However, the multivariate regression models showed that the anthropometric measurements were not significantly correlated with the RSD and HDI.

**Conclusion:**

The current study found an association between the fifth metatarsal intramedullary canal diameter and the fifth metatarsal length and foot anthropometric dimensions. However, the anthropometric measurements of the foot presented a low predictive value for the decision of an intramedullary screw diameter in the treatment of fractures of the base of the fifth metatarsal.

## Introduction

Fractures of the fifth metatarsal base are common, accounting for 61% to 78% of all foot fractures [2]. Still, their operative management poses certain challenges due to the unique anatomic features of the fifth metatarsal and its location. Although most of the fifth metatarsal bone fractures are treated conservatively, a popular method of internal fixation of the fifth metatarsal is by means of a single screw which functions as a form of intramedullary nail [1]. The application of the intramedullary nailing principles on the fifth metatarsal is hampered by the obliquity of its base, the curved shape of its diaphysis and the presence of an isthmus in the medullary canal middle third [5]. Therefore, complications related to this method include breakage of the screw when the fixation is too loose and intraoperative iatrogenic fracture of the diaphysis when the screw purchase is too tight [7]. Last, if the screw is too long, this can lead to straightening of the naturally curved metatarsal and creation of a gap over the fracture site [8, 13].

Specific sets with 0.5 mm increments in the diameter of the screws have been designed to overcome these difficulties. Nevertheless, these sets are not readily available to most primary trauma care centers, where the surgeon relies on the standard small and large fragment fracture sets which have been adopted world-wide. These sets typically include partially- and fully-threaded 3.5 and 4.0 mm screws in the small fragment fracture set and 4.5 and 6.5 mm for the large fragment fracture set respectively. Ideally, the screw thread needs to fit snugly in the medullary canal and anchor its thread in the isthmus region to achieve stability and compression over the fracture site.

Preoperative templating is helpful in intramedullary nailing. In the case of fractures of the base of the fifth metatarsal this becomes less practical, because of the overlap of the lesser metatarsals in the true lateral foot radiograph. An accurate prediction of the suitable screw diameter based on anthropometric measurements. Such a prediction could reduce the rate of intra- and postoperative complications.

Primary aim of the present study was to evaluate the accuracy of anthropometric foot measurements in predicting the diameter of the intramedullary screw for fracture fixation. Secondary aim was to identify whether the fifth metatarsal intramedullary canal diameter is correlated to the fifth metatarsal length and the foot dimensions.

## Materials and methods

Twenty-nine non-paired fresh-frozen cadaveric feet were studied. All specimens were examined to exclude any evidence of injury, defect or surgery before measurement. In each specimen, the maximum length of the plantar surface of the foot and the perimeter at the level of the base of the fifth metatarsal bone were measured using a measuring tape (accuracy 1 mm). Subsequently, the fifth metatarsal of each foot was excised from each foot.

Thereafter, Computed Tomography (CT) scan was performed using a 64 slice MDCT (LightSpeed VCT, GE Healthcare) and a helical mode of acquisition with the tube voltage set to 120 kV and tube current 250 mA. The scanning protocol resulted in images with slice thickness of 0.625 mm, interval 0.625 mm and pitch 0.516:1. Reconstructed images were obtained in an oblique plan according to the longitudinal axis of the bone.

**Fig. 1:**
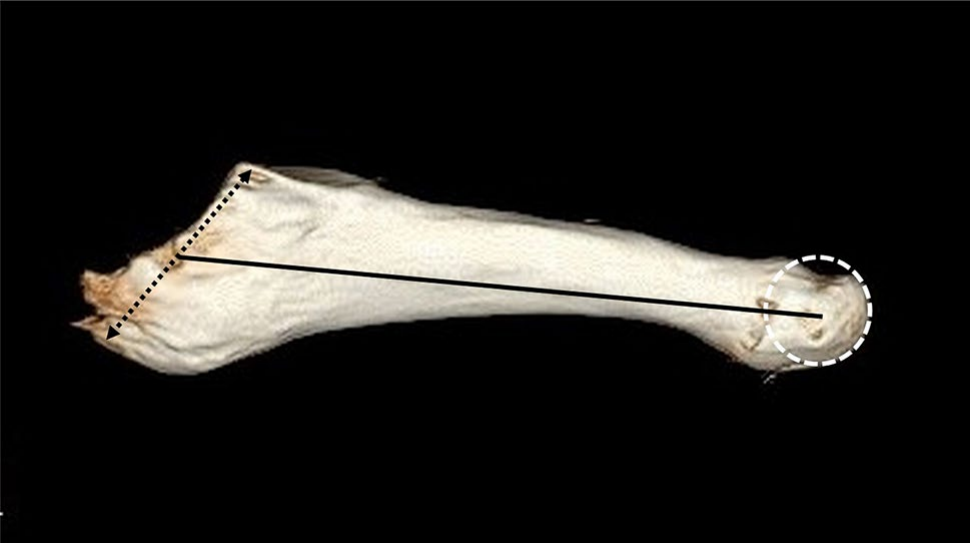
Computed tomography 3D reconstruction image of the fifth metatarsal bone. The midpoint of the metatarsal’s oblique base (black double-headed arrow) was marked as the proximal reference point for length measurement. The midpoint of the fifth metatarsal head transverse diameter (white dotted circle) was defined as the distal reference point for length measurement. The measured metatarsal length (FML) is depicted as the black line

A radiologist specialized in musculoskeletal imaging performed the radiologic measurements. The oblique base of the metatarsal was outlined, and its midpoint was marked as the proximal reference point for length measurement (Fig. 1).

**Fig. 2:**
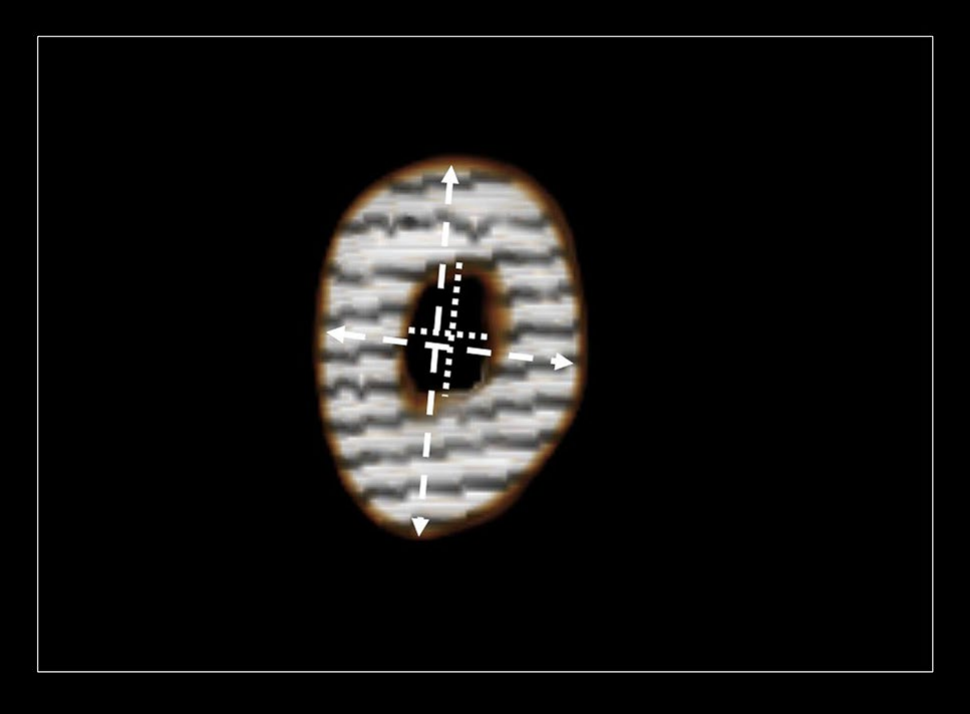
Computed tomography 3D Reconstruction coronal section at isthmus level of the fifth metatarsal bone. The bone’s bowing was defined as the narrowest area of the medullary canal and was used as reference point for isthmus’s measurements. HDI: horizontal diameter of the isthmus (horizontal white dotted line), VDI: vertical diameter of the isthmus (vertical white dotted line), VODI: vertical outer diameter of the isthmus (vertical white double-headed arrow), HODI: horizontal outer diameter of the isthmus (horizontal white double-headed arrow)

The midpoint of the metatarsal head transverse diameter was defined as the distal reference point for length measurement. The metatarsal length was measured and the isthmus position at the level of the bowing of the bone was defined as the narrowest area of the medullary canal. At this level, the horizontal diameter of the isthmus (HDI), the vertical diameter of the isthmus (VDI), the vertical outer diameter of the isthmus (VODI), and the horizontal outer diameter of the isthmus (HODI) were measured (Fig. 2). The HDI values were grouped in 5 mm increments to correspond to the appropriate screw diameter for intramedullary fixation (RSD).

A priori power analysis was performed using the power package in the R software environment. An 80% power and an effect size equal to 0.35, as there were no data from other studies, were set to calculate the sample size. A total of 28 participants was found to be the minimum sample size for the conduction of our research. To identify whether the fifth metatarsal intramedullary canal diameter is correlated to the fifth metatarsal length and the foot dimensions, a univariate linear regression analysis took place which considered RSD and HDI as the dependent variables and FML, PL, PBFM as the independent variables. A multiple regression (linear regression model) was developed to examine whether the anthropometric parameters can be used for a reliable prediction of the diameter of the isthmus. Statistical analysis was performed using IBM SPSS Statistics (Version 27.0. Armonk, NY: IBM Corp). A p-value of less than 0.05 was considered statistically significant.

## Results

The detailed results can be seen in Tables 1–3. In Table 1, the raw parameters data are depicted regarding plantar length (PL), perimeter around the base of the fifth metatarsal bone (PBFM), HDI, VDI, vertical outer diameter of the isthmus (VODI), horizontal outer diameter of the isthmus (HODI), fifth metatarsal bone length (FML), and recommended screw diameter (RSD) for all 29 feet measured. Table 2 demonstrates the mean value, standard deviation (SD) and range of the measurements data.

**Table 1:** Measurements of the anatomic parameters of the cadavers and recommended screws size for each case

No.	PL (cm)	PBFM (cm)	FML (mm)	HDI (mm)	VDI (mm)	HODI (mm)	VODI (mm)	RSD (mm)
1	26.0	20.0	75.0	2.6	5.0	12.0	7.5	3.5
2	24.8	23.9	74.0	4.1	6.4	12.0	8.0	4.0
3	26.8	25.6	76.5	3.0	4.7	12.5	7.2	3.5
4	24.2	22.0	65.0	4.4	5.7	9.0	12.5	4.5
5	28.0	25.5	84.2	6.1	7.5	15.4	9.2	6.0
6	24.4	20.5	76.4	2.6	5.6	9.4	5.5	3.5
7	22.7	20.2	75.0	3.5	6.0	6.6	9.7	3.5
8	24.0	23.0	75.6	3.1	5.6	10.7	8.4	3.5
9	27.8	26.0	81.2	5.7	8.2	14.3	9.5	5.5
10	23.0	21.6	69.1	2.0	2.5	11.1	7.8	3.5
11	23.2	22.0	69.1	5.6	5.3	12.0	8.4	5.5
12	28.0	29.0	89.0	6.1	9.0	15.0	9.2	6.0
13	24.0	23.0	70.0	3.4	6.3	6.8	10.5	3.5
14	26.0	26.0	72.0	3.6	5.0	5.7	8.5	3.5
15	24.5	24.0	78.0	4.1	6.5	7.3	10.1	4.0
16	27.0	25.0	75.0	3.9	5.4	8.1	10.7	4.0
17	27.0	26.0	78.0	6.3	9.4	7.9	13.0	6.5
18	26.0	28.5	76.0	3.6	7.3	6.4	10.4	3.5
19	25.0	21.0	78.0	3.5	5.8	5.4	9.2	3.5
20	25.0	25.5	70.0	4.6	7.0	6.4	10.9	4.5
21	27.0	26.0	77.0	3.8	7.1	6.1	9.8	4.0
22	22.5	22.0	69.0	3.3	6.1	9.1	9.6	3.5
23	25.2	25.5	76.0	4.3	6.5	7.3	9.3	4.5
24	24.6	25.6	70.0	4.4	6.7	8.3	12.4	4.5
25	25.8	25.1	83.0	4.8	7.7	7.5	11.4	5.0
26	25.1	26.0	75.0	3.8	8.0	6.8	12.5	4.0
27	26.7	26.4	83.0	5.4	7.9	7.6	11.1	5.5
28	25.3	24.1	79.0	6.4	7.8	8.9	10.7	6.5
29	24.4	20.4	70.0	4.5	6.5	7.8	10.7	4.5

*N* Number of cadaveric foots, *PL* Plantar length, *PBFM* Perimeter around the base of the fifth metatarsal bone, *VDI* Vertical diameter of the isthmus, *HDI* Horizontal diameter of the isthmus, *VODI* Vertical outer diameter of the isthmus, *HODI* Horizontal outer diameter of the isthmus, *FML* Fifth metatarsal bone length, *RSD* Recommended screw diameter

The HDI values were grouped in 5 mm increments to correspond to the recommended screw diameter for intramedullary fixation

**Table 2:** Descriptive statistics for the measurements of the anatomic parameters of the cadavers

Measurements	*n*	Mean	SD	Minimum	Maximum
PL	29	25.3	1.55	22.5	28.0
PBFM	29	24.1	2.46	20.0	29.0
FML	29	75.5	5.38	65.0	89.0
HDI	29	4.22	1.18	2.00	6.40
VDI	29	6.50	1.42	2.50	9.40
HODI	29	9.08	2.85	5.40	15.40
VODI	29	9.78	1.75	5.50	13.0

PL and PBFM are expressed in cm, while FML, VDI, HDI, VODI, and HODI are expressed in mm

*PL* Plantar length, *PBFM* Perimeter around the base of the fifth metatarsal bone, *VDI* Vertical diameter of the isthmus, *HDI* Horizontal diameter of the isthmus, *VODI* Vertical outer diameter of the isthmus, *HODI* Horizontal outer diameter of the isthmus, *FML* Fifth metatarsal bone length, *n* Number of measurements

In all of the six univariate analyses, it was found that the dependent variable was significantly correlated with the independent variable (RSD vs. FML *p* = 0.007, RSD vs. PBFM *p* = 0.031, RSD vs. PL *p* = 0.009, HDI vs. FML *p* = 0.010, HDI vs. PBFM *p* = 0.010, HDI vs. PL *p* = 0.008) with *p* values < 0.05 (Table 3). The multivariate regression models for anthropometric measurements showed that none of them were significantly correlated with the RSD and HDI (RSD vs. PL *p* = 0.123, RSD vs. PBFM *p* = 0.573, HDI vs. PL *p* = 0.213, HDI vs. PBFM *p* = 0.274) as all *p* values were > 0.05 (Table 3). Specifically, PL and PBFM contribute to a low predictive value for RSD (*R*^*2*^ = 0.235) and HDI (*R*^*2*^ = 0.266), respectively.

**Table 3:** Univariate and multivariate linear regression analysis using the measurements of the anatomic parameters of the cadavers and the recommended screws size

Variables	R^2^	Estimate	95% Confidence interval		*p* values
			Lower	Upper	
*Univariate linear regression*					
RSD vs. FML	0.238	0.090	0.026	0.154	0.007
RSD vs. PL	0.225	0.304	0.081	0.526	0.009
RSD vs. PBFM	0.160	0.162	0.016	0.308	0.031
HDI vs. FML	0.222	0.103	0.027	0.180	0.010
HDI vs. PL	0.230	0.365	0.101	0.628	0.008
HDI vs. PBFM	0.220	0.225	0.058	0.392	0.010
*Multivariate linear regression*					
RSD	0.235				
*vs. PL*		0.243	−0.071	0.557	0.123
*vs. PBFM*		0.055	−0.143	0.254	0.573
HDI	0.266				
*vs. PL*		0.227	−0.138	0.592	0.213
*vs. PBFM*		0.125	−0.105	0.356	0.274

Estimate refers to the difference between dependent and independent variables’ values. RSD and HDI are the dependent variables of the analysis. PL, RBFM and FML are the independent variables

*PL* Plantar length, *PBFM* Perimeter around the base of the fifth metatarsal bone, *HDI* Horizontal diameter of the isthmus, *RSD* Recommended screw diameter, *FML* Fifth metatarsal bone length, *vs* versus

## Discussion

Intramedullary fixation by means of a single screw remains a popular way of operative management in fractures of the base of the fifth metatarsal, especially those involving the articulation of the fourth-fifth intermetatarsal facet [3]. According to the anatomic zone classification by Lawrence and Botte, these Zone 2 fractures are best treated surgically to avoid the considerable risk of non-union, whose incidence has been reported as high as 21% [4, 9]. On the other hand, the optimal diameter of the screw has been a matter of considerable debate. Basic principles dictate that the largest screw possible which will achieve maximal contact interface with the dense cortical bone should always be used [4]. However, according to different studies this diameter varies from 4.5 to 5.5 mm [5]. Fifth metatarsal length measured with CT in the present study was correlated with the HDI and RSD. However, templating of the screw size based on preoperative radiographs of the uninjured side can lead to errors, because plain radiographs tend to overestimate the metatarsal length and underestimate the medullary canal width [5]. In the current study, the length of the fifth metatarsal was not a reliable predictor of the optimal screw diameter. Therefore, obtaining a radiograph of the contralateral, uninjured side may not be useful for templating in the treatment of fifth metatarsal fractures.

Anthropometric measurements of the foot could be used as a predictor of the optimal screw diameter. The relation of anthropometric measurements to the characteristics of the fifth metatarsal have been studied before. DeSandis et al. found a positive correlation of the patient’s height and weight to the length the fifth metatarsal and the medullary canal width [5]. In the present study, although the univariate analysis demonstrated significant correlation between both anthropometric measurements (PL and PBFM) and the HDI and RSD, the multivariate analysis showed no significant correlation. A possible explanation is that the anthropometric size is normally correlated with the size of the medullary canal, but the predictive value of those measurement is low (23.5% for the RSD and 26.6 for the HDI). The intramedullary canal of the fifth metatarsal is elliptical in shape. In most studies the VDI is greater than the HDI. In imaging of isolated metatarsals which are excised from cadavers the true reference of the VDI and HDI axes can be altered when compared to CTs of the whole foot [10]. This has led to conflicting findings in the past [6]. Therefore, for the purposes of this study care was taken to properly place the metatarsals and identify the respective axes correctly. The narrower horizontal (mediolateral) diameter (HDI) was chosen to predict the maximum screw diameter.

Several previous cadaveric studies have aimed to optimize the prediction of the ideal screw diameter for intramedullary fixation of the fifth MT. Scott et al. used a digital caliper tο measure the maximum coronal and axial diameters at the level of the isthmus in 25 transected metatarsals. They found a mean dorsal to plantar diameter of 6.475 ± 1.54 (range 4 to 12) mm and a mean medial to lateral diameter measured 4.6 ± 0.85 (range 3 to 6) mm. The authors suggested a 4.5 mm cannulated screw as the narrowest diameter of screw that could be appropriate for the fifth metatarsal. No CT was used in their study [11]. In the largest relevant study, Ochenjele et al. studied 119 MTs with CT. They measured the medullary canal at the bow of the metatarsal and a point 40 mm from the base of the fifth metatarsal, according to the usual fracture location and the respective necessary screw length for adequate mechanical stability. The dorsal to plantar medullary diameter was 6.7 mm (range 4.0–9.3) at the bow and 7.0 mm (range 4.0–10.5) at the 40 mm point. The medial to lateral diameter was 5.0 mm (range 3.1–8.0) at the bow and 5.1 mm (range 3.0–7.5) at the 40 mm point respectively [10]. What is noteworthy in the above studies and is also found in our measurements is the relatively wide range of the medullary canal dimensions among different individuals. The clinical relevance of this is that no single screw diameter can be suitable for the majority of patients with a fifth metatarsal fracture.

A relative weakness of the study is that the gender of the cadavers was not recorded. Gender differences in the length of the fifth metatarsal have been recorded, with a median length of 7.4 cm in males versus 6.79 cm in females [12]. However, previous studies comparing coronal canal diameter between male and female patients showed no statistical differences [5, 10]. Foot surgeons should keep in mind that larger individuals could present more bowing in their metatarsal shaft which may affect the selection of the proper RSD. Moreover, in real-life scenario, anthropometric measurements (e.g., PBFM and PL) in a fractured foot may be challenging in cases with marked foot swelling.

## Conclusion

The detailed morphometric documentation of the metatarsal anatomy in relation to anthropometric measurements provides clinically relevant data for future research. The current study found an association between the fifth metatarsal intramedullary canal diameter and the fifth metatarsal length and foot anthropometric dimensions. However, the anthropometric measurements of the foot presented a low predictive value for the decision of an intramedullary screw diameter in the treatment of fractures of the base of the fifth metatarsal.

## Data Availability

Not applicable.

## References

[1] Biz C, Zamperetti M, Gasparella A (2017). Early radiographic and clinical outcomes of minimally displaced proximal fifth metatarsal fractures: cast vs functional bandage. Muscles Ligaments Tendons J.

[2] Bušková K, Bartoníček J, Rammelt S (2021). Fractures of the base of the fifth metatarsal bone: a critical analysis review. JBJS Rev.

[3] Cheung CN, Lui TH (2016). Proximal fifth metatarsal fractures: anatomy, classification, treatment and complications. Arch Trauma Res.

[4] Chloros GD, Kakos CD, Tastsidis IK (2022). Fifth metatarsal fractures: an update on management, complications, and outcomes. EFORT Open Rev.

[5] DeSandis B, Murphy C, Rosenbaum A (2016). Multiplanar CT analysis of fifth metatarsal morphology: implications for operative management of zone II fractures. Foot Ankle Int.

[6] Ebraheim NA, Haman SP, Lu J (2000). Anatomical and radiological considerations of the fifth metatarsal bone. Foot Ankle Int.

[7] Horst F, Gilbert BJ, Glisson RR, Nunley JA (2004). Torque resistance after fixation of Jones fractures with intramedullary screws. Foot Ankle Int.

[8] Kelly IP, Glisson RR, Fink C (2001). Intramedullary screw fixation of Jones fractures. Foot Ankle Int.

[9] Lawrence SJ, Botte MJ (1993). Jones’ fractures and related fractures of the proximal fifth metatarsal. Foot Ankle.

[10] Ochenjele G, Ho B, Switaj PJ (2015). Radiographic study of the fifth metatarsal for optimal intramedullary screw fixation of Jones fracture. Foot Ankle Int.

[11] Scott RT, Hyer CF, DeMill SL (2015). Screw fixation diameter for fifth metatarsal jones fracture: a cadaveric study. J Foot Ankle Surg Off Publ Am Coll Foot Ankle Surg.

[12] Senol D, Bodur F, Seçgin Y (2022). Sex prediction with morphometric measurements of first and fifth metatarsal and phalanx obtained from X-ray images by using machine learning algorithms. Folia Morphol.

[13] Shah SN, Knoblich GO, Lindsey DP (2001). Intramedullary screw fixation of proximal fifth metatarsal fractures: a biomechanical study. Foot Ankle Int.

